# Supplementary material for: Production of human translation-competent lysates using dual centrifugation
Source: RNA Biol. 2021 Dec 29;19(1):78–88. doi: 10.1080/15476286.2021.2014695 (PMC8815625; doi:10.1080/15476286.2021.2014695)
Supplement: Supplemental Material [file KRNB_A_2014695_SM9599.zip › supplementary/2021.11.26 Revised sup. material.docx]

**Production of human translation-competent lysates using dual centrifugation**

Lukas-Adrian Gurzeler, Jana Ziegelmüller, Oliver Mühlemann and Evangelos D. Karousis

**SUPPLEMENTARY MATERIAL**


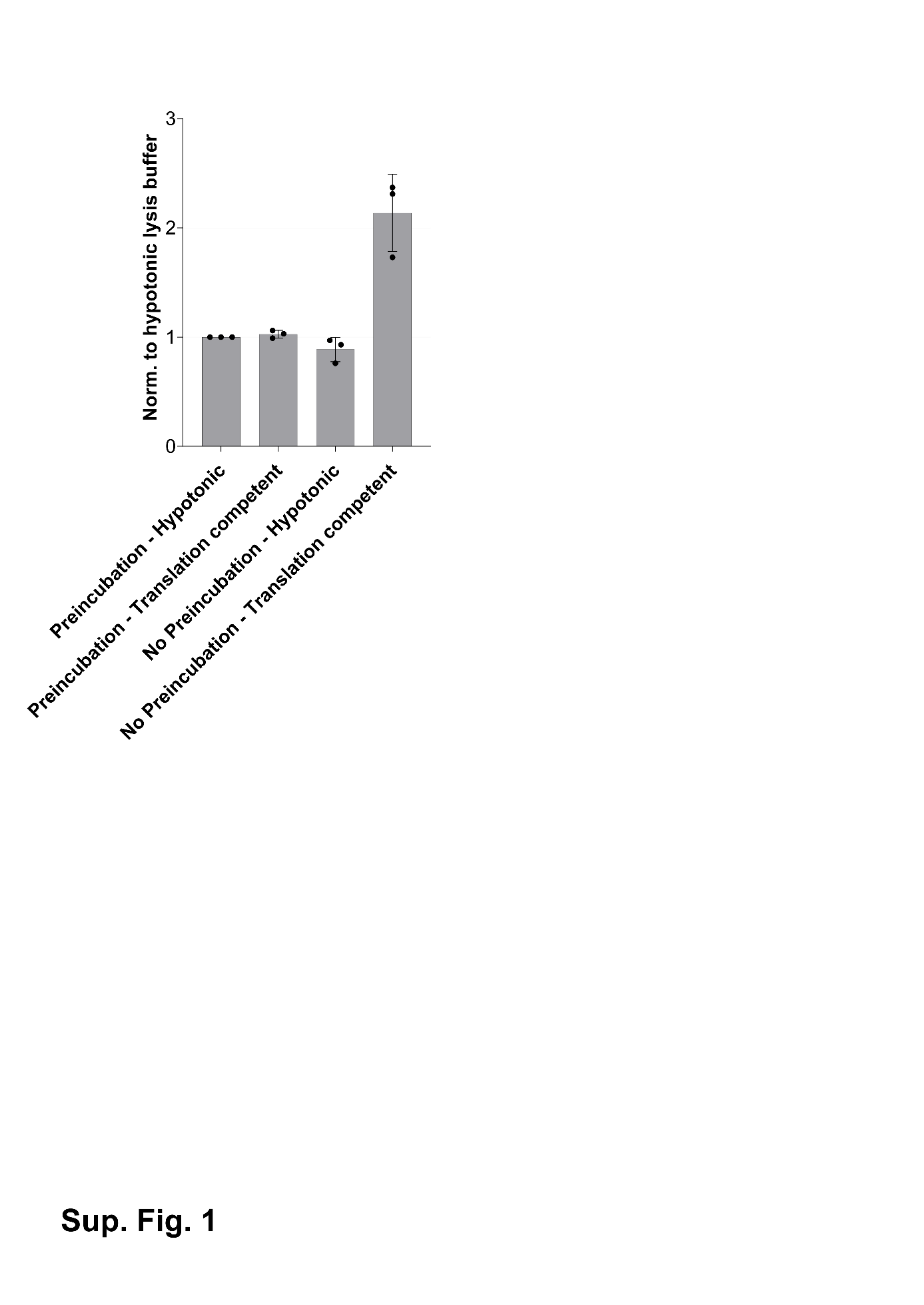


**Sup. Fig. 1:** Comparison of lysates produced using a hypotonic lysis buffer complemented with components required for *in vitro* translation (hypotonic) versus lysis in a translation-competent buffer (translation comp lysis) with or without a 5΄ pre-incubation at 33 °C before *in vitro* translation. RLuc activity measurements are depicted as normalized values of luminescence to the hypotonic buffer condition with pre-incubation. Each dot depicts the value of an individual experiment and *in vitro* translation (biological replicate) that was measured three times (technical replicates). *In vitro* translation experiments were performed using 10 fmol/μl RLuc reporter. Mean values and SD are shown.

**
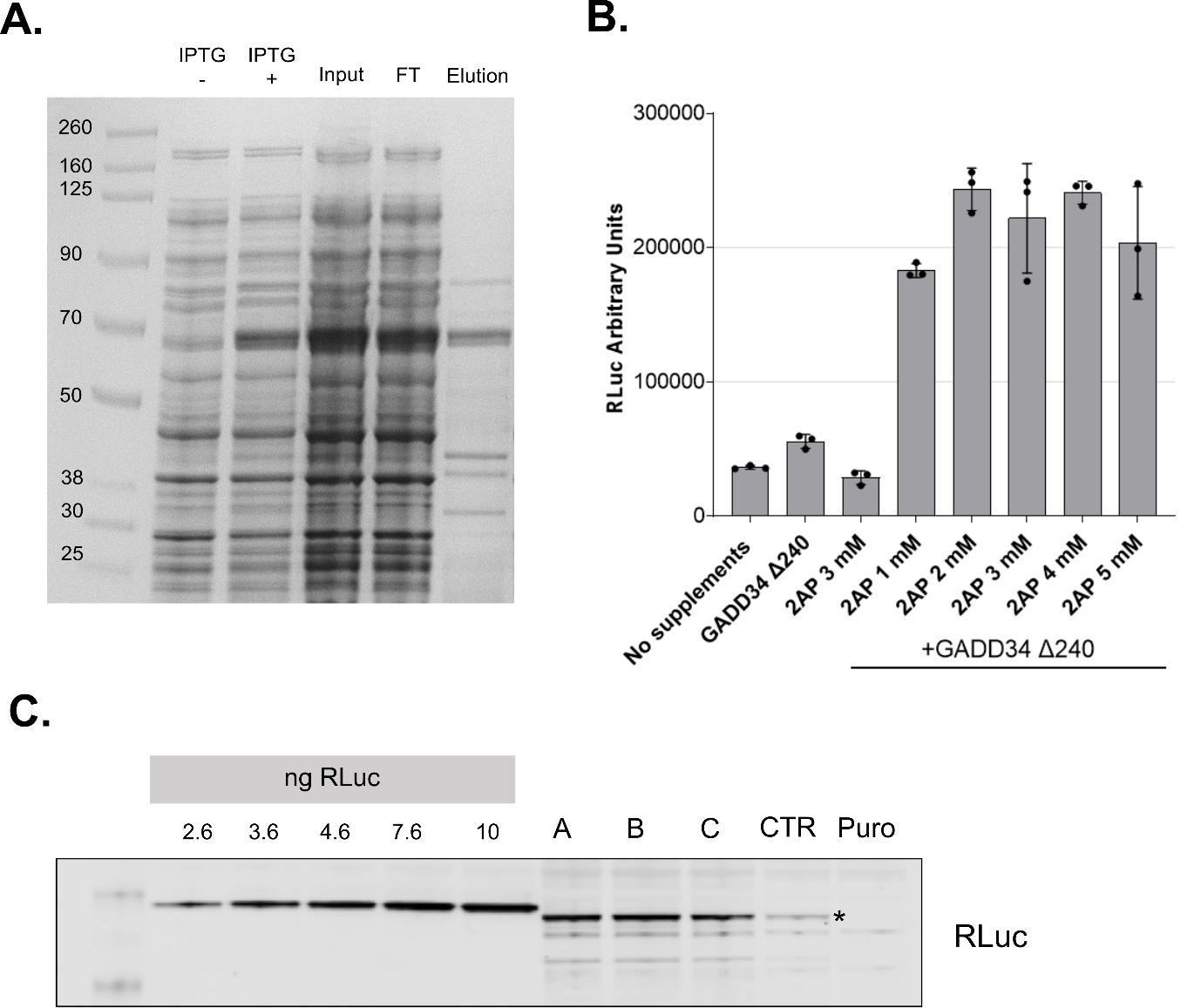
**

**Sup. Fig. 2: A.** Coomassie-stained gel showing the purification steps of GADD34 Δ240. Bacterial extract in the presence or absence of IPTG in the culture. Samples (5 μl) of input, flowthrough and purified GADD34-His fractions were analyzed. **B.** Comparison of RLuc measurements of *in vitro* translation reactions of DC-treated lysates in the absence (No supplements) or the presence of 0.5 mM GADD34 Δ240, 3 mM 2-aminopurine and increasing concentrations of 2-aminopurine in the presence of 0.5 mM GADD34 Δ240. RLuc activity measurements are depicted as arbitrary units (AU) of luminescence. The dots depict the values of three individual experiments (biological replicates) that were measured each three times (technical replicates). **C.** Western blot analysis of increasing amounts of recombinant RLuc diluted in translation competent and lysates corresponding to 6.67x10^5^ cells after *in vitro* translation against RLuc of non supplemented lysate (CTR), or three replicates (A, B, and C) of lysates supplemented with 0.5 mM GADD34 Δ240 and 3 mM 2-aminopurine. *In vitro* translation experiments were performed using 10 fmol/μl RLuc reporter without preincubating the translation reactions before adding the reporter mRNAs.

**Sup. Fig. 3**

Nucleotide sequences of the DNA templates used for in vitro transcription followed by the colour code for every sequence. Start and stop codons are in bolt and underlined.

**3.1 DNA template for hRLuc mRNA reporter (from pCRII_ p200-6xMS2)**

TAATACGACTCACTATAGGGCGAATTGGGCCCTCTAGATGCATGCTCGAGCGGCCGCCAGTGTGATGGATATCTGCAGAATTCGCCCTTC**ATG**GCTTCCAAGGTGTACGACCCCGAGCAACGCAAACGCATGATCACTGGGCCTCAGTGGTGGGCTCGCTGCAAGCAAATGAACGTGCTGGACTCCTTCATCAACTACTATGATTCCGAGAAGCACGCCGAGAACGCCGTGATTTTTCTGCATGGTAACGCTGCCTCCAGCTACCTGTGGAGGCACGTCGTGCCTCACATCGAGCCCGTGGCTAGATGCATCATCCCTGATCTGATCGGAATGGGTAAGTCCGGCAAGAGCGGGAATGGCTCATATCGCCTCCTGGATCACTACAAGTACCTCACCGCTTGGTTCGAGCTGCTGAACCTTCCAAAGAAAATCATCTTTGTGGGCCACGACTGGGGGGCTTGTCTGGCCTTTCACTACTCCTACGAGCACCAAGACAAGATCAAGGCCATCGTCCATGCTGAGAGTGTCGTGGACGTGATCGAGTCCTGGGACGAGTGGCCTGACATCGAGGAGGATATCGCCCTGATCAAGAGCGAAGAGGGCGAGAAAATGGTGCTTGAGAATAACTTCTTCGTCGAGACCATGCTCCCAAGCAAGATCATGCGGAAACTGGAGCCTGAGGAGTTCGCTGCCTACCTGGAGCCATTCAAGGAGAAGGGCGAGGTTAGACGGCCTACCCTCTCCTGGCCTCGCGAGATCCCTCTCGTTAAGGGAGGCAAGCCCGACGTCGTCCAGATTGTCCGCAACTACAACGCCTACCTTCGGGCCAGCGACGATCTGCCTAAGATGTTCATCGAGTCCGACCCTGGGTTCTTTTCCAACGCTATTGTCGAGGGAGCTAAGAAGTTCCCTAACACCGAGTTCGTGAAGGTGAAGGGCCTCCACTTCAGCCAGGAGGACGCTCCAGATGAAATGGGTAAGTACATCAAGAGCTTCGTGGAGCGCGTGCTGAAGAACGAGCAG**TAA**TTCTAGAGGATCATAATCAGCCATACCACATTTGTAGAGGTTTTACTTGCTTTAAAAAACCTCCCACACCTCCCCCTGAACCTGAAACATAAAATGAATGCAATTGTTGTTGTTAACTTGTTTATTGCAGCTTATAATGGTTACAAATAAAGCAATAGCATCACCATAATAACATATGGTTCCCTAAGTCCAACTACCAAACTGGGTCTAGCTCTAGCTGTAGAAAACATGAGGATCACCCATGTCTGCTGGACGACTGTAGAAAACATGAGGATCACCCATGTCTGCTGTCTAGCTGTAGAAAACATGAGGATCACCCATGTCTGCTGGACGACTGTAGAAAACATGAGGATCACCCATGTCTGCTGTCTAGCTGTAGAAAACATGAGGATCACCCATGTCTGCTGGACGACTGTAGAAAACATGAGGATCACCCATGTCTGCTGTCTAGGGtcgacaatcaacctctggGAGCTCCTGCCTCGAGCTTCCTCATCGCCGGTACCAAAAAAAAAAAAAAAAAAAAAAAAAAAAAAAAAAAAAAAAAAAAAAAAAAAAAAAAAAAAAAAAAAAAAAAAAAAAAAAATTTTTGGAAAAGCTT

|  | T7 Promoter |  | MS2 binding sites |
| --- | --- | --- | --- |
|  |  |  |  |
|  | 5’UTR |  | A(80) |
|  |  |  |  |
|  | hRLuc coding sequence |  | *Hind*III recognition site |
|  |  |  |  |
|  | 3’UTR |  |  |
|  |  |  |  |

**3.2 DNA template for 3xFlag-Beta Globin (HBB) mRNA reporter** (from pCRII_ SC-LD _3xFLAG-HBB-CTE_A80)

TAATACGACTCACTATAGATTAAAGGTTTATACCTTCCCAGGTAACAAACCAACCAACTTTCGATCTCTTGTAGATCTGTTCTCTAAACG**ATG**GACTACAAGGACCACGACGGTGACTACAAGGACCACGACATCGACTACAAGgacgacgacgacaagctcggatcaatggtgcacctgactcctgaggagaagtctgccgttactgccctgtggggcaaggtgaacgtggatgaagttggtggtgaggccctgggcaggctgctggtggtctacccttggacccagaggttctttgagtcctttggggatctgtccactcctgatgctgttatgggcaaccctaaggtgaaggctcatggcaagaaagtgctcggtgcctttagtgatggcctggctcacctggacaacctcaagggcacctttgccacactgagtgagctgcactgtgacaagctgcacgtggatcctgagaacttcaggctcctgggcaacgtgctggtctgtgtgctggcccatcactttggcaaagaattcaccccaccagtgcaggctgcctatcagaaagtggtggctggtgtggctaatgccctggcccacaagtatcacatggaaccgctggtgctgagtgcgaaaaaactgagcagcctgctgacctgcaaatatattgcggcg**taa**aTTCTAGAGGATCATAATCAGCCATACCACATTTGTAGAGGTTTTACTTGCTTTAAAAAACCTCCCACACCTCCCCCTGAACCTGAAACATAAAATGAATGCAATTGTTGTTGTTAACTTGTTTATTGCAGCTTATAATGGTTACAAATAAAGCAATAGCATCACAAATTTCACAAATAAAGCATTTTTTTCACTGCCTCGAGCTTCCTCATCGCCGGTACCAAAAAAAAAAAAAAAAAAAAAAAAAAAAAAAAAAAAAAAAAAAAAAAAAAAAAAAAAAAAAAAAAAAAAAAAAAAAAAAATTTTTGGAAAAGCTT

|  | T7 Promoter (mini) |  | C-terminal extension |
| --- | --- | --- | --- |
|  |  |  |  |
|  | SarsCov2 Leader |  | 3’UTR |
|  |  |  |  |
|  | 3xFLAG |  | A(80) |
|  |  |  |  |
|  | Beta Globin coding sequence |  | *Hind*III recognition site |
|  |  |  |  |
|  |  |  |  |

**3.2 DNA template for dual luciferase mRNA reporter (from pCRII-RLuc-UAA-FLuc-A80)**

TAATACGACTCACTATAGGGCGAATTGGGCCCTCTAGATGCATGCTCGAGCGGCCGC**atg**acttcgaaagtttatgatccagaacaaaggaaacggatgataactggtccgcagtggtgggccagatgtaaacaaatgaatgttcttgattcatttattaattattatgattcagaaaaacatgcagaaaatgctgttatttttttacatggtaacgcggcctcttcttatttatggcgacatgttgtgccacatattgagccagtagcgcggtgtattataccagaccttattggtatgggcaaatcaggcaaatctggtaatggttcttataggttacttgatcattacaaatatcttactgcatggtttgaacttcttaatttaccaaagaagatcatttttgtcggccatgattggggtgcttgtttggcatttcattatagctatgagcatcaagataagatcaaagcaatagttcacgctgaaagtgtagtagatgtgattgaatcatgggatgaatggcctgatattgaagaagatattgcgttgatcaaatctgaagaaggagaaaaaatggttttggagaataacttcttcgtggaaaccatgttgccatcaaaaatcatgagaaagttagaaccagaagaatttgcagcatatcttgaaccattcaaagagaaaggtgaagttcgtcgtccaacattatcatggcctcgtgaaatcccgttagtaaaaggtggtaaacctgacgttgtacaaattgttaggaattataatgcttatctacgtgcaagtgatgatttaccaaaaatgtttattgaatcggacccaggattcttttccaatgctattgttgaaggtgccaagaagtttcctaatactgaatttgtcaaagtaaaaggtcttcatttttcgcaagaagatgcacctgatgaaatgggaaaatatatcaaatcgttcgttgagcgagttctcaaaaatgaacaaatgtcgacggtctccctccactgctgtagtaacccgggtccggggcctcggtggtgc**taa**g**atg**cccctcacccacccctgaagatcccaggtgggcgagggaatagtcagagggatcacaatctttcagctggatccttcaacttccctgagctcgaagacgccaaaaacataaagaaaggcccggcgccattctatcctctagaggatggaaccgctggagagcaactgcataaggctatgaagagatacgccctggttcctggaacaattgcttttacagatgcacatatcgaggtgaacatcacgtacgcggaatacttcgaaatgtccgttcggttggcagaagctatgaaacgatatgggctgaatacaaatcacagaatcgtcgtatgcagtgaaaactctcttcaattctttatgccggtgttgggcgcgttatttatcggagttgcagttgcgcccgcgaacgacatttataatgaacgtgaattgctcaacagtatgaacatttcgcagcctaccgtagtgtttgtttccaaaaaggggttgcaaaaaattttgaacgtgcaaaaaaaattaccaataatccagaaaattattatcatggattctaaaacggattaccagggatttcagtcgatgtacacgttcgtcacatctcatctacctcccggttttaatgaatacgattttgtaccagagtcctttgatcgtgacaaaacaattgcactgataatgaattcctctggatctactgggttacctaagggtgtggcccttccgcatagaactgcctgcgtcagattctcgcatgccagagatcctatttttggcaatcaaatcattccggatactgcgattttaagtgttgttccattccatcacggttttggaatgtttactacactcggatatttgatatgtggatttcgagtcgtcttaatgtatagatttgaagaagagctgtttttacgatcccttcaggattacaaaattcaaagtgcgttgctagtaccaaccctattttcattcttcgccaaaagcactctgattgacaaatacgatttatctaatttacacgaaattgcttctgggggcgcacctctttcgaaagaagtcggggaagcggttgcaaaacgcttccatcttccagggatacgacaaggatatgggctcactgagactacatcagctattctgattacacccgagggggatgataaaccgggcgcggtcggtaaagttgttccattttttgaagcgaaggttgtggatctggataccgggaaaacgctgggcgttaatcagagaggcgaattatgtgtcagaggacctatgattatgtccggttatgtaaacaatccggaagcgaccaacgccttgattgacaaggatggatggctacattctggagacatagcttactgggacgaagacgaacacttcttcatagttgaccgcttgaagtctttaattaaatacaaaggatatcaggtggcccccgctgaattggaatcgatattgttacaacaccccaacatcttcgacgcgggcgtggcaggtcttcccgacgatgacgccggtgaacttcccgccgccgttgttgttttggagcacggaaagacgatgacggaaaaagagatcgtggattacgtcgccagtcaagtaacaaccgcgaaaaagttgcgcggaggagttgtgtttgtggacgaagtaccgaaaggtcttaccggaaaactcgacgcaagaaaaatcagagagatcctcataaaggccaagaagggcggaaagtccaaattg**taa**GGTACCAAAAAAAAAAAAAAAAAAAAAAAAAAAAAAAAAAAAAAAAAAAAAAAAAAAAAAAAAAAAAAAAAAAAAAAAAAAAAAAATTTTTGGAAAAGCTT

|  | T7 Promoter |  | FLuc coding sequence |
| --- | --- | --- | --- |
|  |  |  |  |
|  | 5’UTR |  | A(80) |
|  |  |  |  |
|  | RLuc coding sequence |  | *Hind*III recognition site |
|  |  |  |  |

**3.2 DNA template for dual luciferase mRNA reporter with IRES (from pCRII-RLuc-IRES-FLuc-A80)**

TAATACGACTCACTATAGGGCGAATTGGGCCCTCTAGATGCATGCTCGAGCGGCCGC**atg**acttcgaaagtttatgatccagaacaaaggaaacggatgataactggtccgcagtggtgggccagatgtaaacaaatgaatgttcttgattcatttattaattattatgattcagaaaaacatgcagaaaatgctgttatttttttacatggtaacgcggcctcttcttatttatggcgacatgttgtgccacatattgagccagtagcgcggtgtattataccagaccttattggtatgggcaaatcaggcaaatctggtaatggttcttataggttacttgatcattacaaatatcttactgcatggtttgaacttcttaatttaccaaagaagatcatttttgtcggccatgattggggtgcttgtttggcatttcattatagctatgagcatcaagataagatcaaagcaatagttcacgctgaaagtgtagtagatgtgattgaatcatgggatgaatggcctgatattgaagaagatattgcgttgatcaaatctgaagaaggagaaaaaatggttttggagaataacttcttcgtggaaaccatgttgccatcaaaaatcatgagaaagttagaaccagaagaatttgcagcatatcttgaaccattcaaagagaaaggtgaagttcgtcgtccaacattatcatggcctcgtgaaatcccgttagtaaaaggtggtaaacctgacgttgtacaaattgttaggaattataatgcttatctacgtgcaagtgatgatttaccaaaaatgtttattgaatcggacccaggattcttttccaatgctattgttgaaggtgccaagaagtttcctaatactgaatttgtcaaagtaaaaggtcttcatttttcgcaagaagatgcacctgatgaaatgggaaaatatatcaaatcgttcgttgagcgagttctcaaaaatgaacaaatgtcgacggtctccctccactgctgtagtaacccgggtccggggcctcggtggtgc**taa**GCGAATTAATTCCGGTTATTTTCCACCATATTGCCGTCTTTTGGCAATGTGAGGGCCCGGAAACCTGGCCCTGTCTTCTTGACGAGCATTCCTAGGGGTCTTTCCCCTCTCGCCAAAGGAATGCAAGGTCTGTTGAATGTCGTGAAGGAAGCAGTTCCTCTGGAAGCAACTTGAAGACAAACAACGTCTGTAGCGACCCTTTGCAGGCAGCGGAACCCCCCACCTGGCGACAGGTGCCTCTGCGGCCAAAAGCCACGTGTATAAGATACACCTGCAAAGGCGGCACAACCCCAGTGCCACGTTGTGAGTTGGATAGTTGTGGAAAGAGTCAAATGGCTCACCTCAAGCGTATTCAACAAGGGGCTGAAGGATGCCCAGAAGGTACCCCATTGTATGGGATCTGATCTGGGGCCTCGGTGCACATGCTTTACATGTGTTTAGTCGAGGTTAAAAAACGTCTAGGCCCCCCGAACCACGGGGACGTGGTTTTCCTTTGAAAAACACGATGATAAT**ATG**gaagacgccaaaaacataaagaaaggcccggcgccattctatcctctagaggatggaaccgctggagagcaactgcataaggctatgaagagatacgccctggttcctggaacaattgcttttacagatgcacatatcgaggtgaacatcacgtacgcggaatacttcgaaatgtccgttcggttggcagaagctatgaaacgatatgggctgaatacaaatcacagaatcgtcgtatgcagtgaaaactctcttcaattctttatgccggtgttgggcgcgttatttatcggagttgcagttgcgcccgcgaacgacatttataatgaacgtgaattgctcaacagtatgaacatttcgcagcctaccgtagtgtttgtttccaaaaaggggttgcaaaaaattttgaacgtgcaaaaaaaattaccaataatccagaaaattattatcatggattctaaaacggattaccagggatttcagtcgatgtacacgttcgtcacatctcatctacctcccggttttaatgaatacgattttgtaccagagtcctttgatcgtgacaaaacaattgcactgataatgaattcctctggatctactgggttacctaagggtgtggcccttccgcatagaactgcctgcgtcagattctcgcatgccagagatcctatttttggcaatcaaatcattccggatactgcgattttaagtgttgttccattccatcacggttttggaatgtttactacactcggatatttgatatgtggatttcgagtcgtcttaatgtatagatttgaagaagagctgtttttacgatcccttcaggattacaaaattcaaagtgcgttgctagtaccaaccctattttcattcttcgccaaaagcactctgattgacaaatacgatttatctaatttacacgaaattgcttctgggggcgcacctctttcgaaagaagtcggggaagcggttgcaaaacgcttccatcttccagggatacgacaaggatatgggctcactgagactacatcagctattctgattacacccgagggggatgataaaccgggcgcggtcggtaaagttgttccattttttgaagcgaaggttgtggatctggataccgggaaaacgctgggcgttaatcagagaggcgaattatgtgtcagaggacctatgattatgtccggttatgtaaacaatccggaagcgaccaacgccttgattgacaaggatggatggctacattctggagacatagcttactgggacgaagacgaacacttcttcatagttgaccgcttgaagtctttaattaaatacaaaggatatcaggtggcccccgctgaattggaatcgatattgttacaacaccccaacatcttcgacgcgggcgtggcaggtcttcccgacgatgacgccggtgaacttcccgccgccgttgttgttttggagcacggaaagacgatgacggaaaaagagatcgtggattacgtcgccagtcaagtaacaaccgcgaaaaagttgcgcggaggagttgtgtttgtggacgaagtaccgaaaggtcttaccggaaaactcgacgcaagaaaaatcagagagatcctcataaaggccaagaagggcggaaagtccaaattg**taa**GGTACCAAAAAAAAAAAAAAAAAAAAAAAAAAAAAAAAAAAAAAAAAAAAAAAAAAAAAAAAAAAAAAAAAAAAAAAAAAAAAAAATTTTTGGAAAAGCTT

|  | T7 Promoter (mini) |  | C-terminal extension |
| --- | --- | --- | --- |
|  |  |  |  |
|  | 5’UTR |  | A(80) |
|  |  |  |  |
|  | RLuc coding sequence |  | *Hind*III recognition site |
|  |  |  |  |
|  | ECMV IRES |  |  |
|  |  |  |  |
|  |  |  |  |

**3.4 DNA template for SMG6-3xFlag mRNA reporter** (from pCRII_ SC-LD_SMG6-3xFlag_A80)

TAATACGACTCACTATAGATTAAAGGTTTATACCTTCCCAGGTAACAAACCAACCAACTTTCGATCTCTTGTAGATCTGTTCTCTAAACG**ATG**GCGGAAGGGCTGGAGCGTGTGCGGATCTCCGCGTCGGAGCTGCGCGGGATCCTGGCTACTCTGGCCCCGCAGGCCGGGAGCAGAGAAAACATGAAGGAATTAAAGGAGGCCAGGCCGCGCAAAGATAACAGGCGTCCAGATCTGGAAATCTATAAGCCTGGCCTTTCTCGGCTAAGGAACAAGCCCAAAATCAAGGAACCCCCTGGGAGTGAGGAATTCAAAGATGAAATTGTTAATGACCGAGATTGCTCTGCTGTTGAAAATGGTACACAGCCCGTTAAAGATGTCTGCAAGGAACTGAACAACCAAGAGCAGAATGGTCCTATAGACCCAGAAAATAATCGGGGACAAGAATCCTTTCCTAGGACTGCTGGACAAGAGGATCGTAGTCTAAAAATTATCAAAAGAACAAAGAAACCCGACCTGCAGATCTATCAGCCTGGACGACGTTTGCAGACTGTTAGCAAAGAATCCGCCAGTCGGGTGGAGGAGGAAGAAGTCCTCAACCAGGTAGAACAACTGAGAGTAGAGGAAGATGAGTGTAGGGGAAATGTTGCGAAGGAGGAAGTTGCGAATAAACCAGACAGGGCCGAGATAGAAAAGAGCCCAGGTGGTGGGAGAGTAGGGGCTGCAAAAGGAGAAAAAGGAAAGAGGATGGGAAAAGGGGAGGGGGTGAGGGAAACCCACGACGACCCGGCCCGCGGGAGGCCGGGCTCCGCAAAGCGCTACTCCCGCTCAGACAAACGAAGGAATCGCTACCGCACGCGCAGCACCAGCTCAGCTGGCAGCAACAACAGCGCTGAGGGAGCTGGCCTGACGGATAATGGATGTCGCCGCCGCCGACAGGATAGGACCAAGGAGAGGCCACCACTGAAGAAGCAAGTGTCTGTGTCCTCAACCGATTCCTTAGACGAGGACAGAATTGATGAGCCTGATGGATTAGGACCCAGGAGAAGTTCAGAAAGGAAGAGACATTTAGAAAGAAACTGGTCTGGCCGTGGGGAGGGTGAGCAGAAAACCAGTGCTAAAGAATATCGAGGCACTCTTCGTGTCACTTTCGATGCAGAAGCCATGAACAAAGAGTCTCCCATGGTGAGGTCAGCCAGGGATGATATGGATAGAGGAAAGCCTGACAAAGGCTTGAGCAGTGGGGGCAAAGGCTCTGAGAAGCAGGAGTCCAAAAACCCGAAACAAGAACTTCGGGGTCGTGGTCGTGGCATTCTGATTTTGCCTGCCCATACCACCCTATCTGTCAATTCAGCAGGTTCTCCAGAGTCCGCGCCTTTGGGACCTCGGCTTTTGTTTGGATCTGGTAGTAAGGGATCTCGGAGTTGGGGCCGTGGAGGCACCACACGCCGATTGTGGGACCCAAACAATCCTGATCAGAAACCTGCTCTAAAGACTCAGACGCCCCAGCTACATTTCTTGGACACTGATGATGAAGTCAGCCCTACATCTTGGGGTGACTCACGCCAGGCTCAGGCATCTTACTATAAGTTTCAAAACTCTGACAACCCCTATTATTACCCCCGGACACCAGGCCCTGCCTCCCAGTATCCCTATACGGGCTATAACCCTCTACAGTACCCAGTGGGCCCTACGAATGGTGTGTACCCAGGGCCTTACTACCCAGGCTACCCGACTCCGTCAGGACAGTATGTGTGTAGCCCTCTACCTACCAGCACCATGAGTCCCGAGGAGGTAGAGCAGCACATGAGGAACCTGCAGCAACAGGAGCTGCACAGGCTTCTCCGGGTGGCTGACAACCAGGAACTGCAGCTCAGCAACCTGCTCTCCAGGGACCGCATCAGTCCGGAGGGCCTGGAGAAGATGGCGCAACTCAGAGCTGAACTGCTGCAGCTATATGAGCGCTGTATTCTATTAGATATTGAGTTCTCTGATAATCAGAATGTGGATCAGATCCTGTGGAAGAATGCTTTCTATCAGGTGATTGAGAAGTTCAGGCAACTTGTCAAGGATCCGAATGTTGAGAACCCAGAACAGATTCGGAACAGACTTTTGGAGCTCTTGGATGAGGGTAGTGACTTCTTTGATAGTTTGCTTCAGAAGCTCCAAGTTACTTATAAGTTCAAACTGGAAGACTACATGGATGGTCTTGCCATTCGCAGCAAGCCATTACGCAAGACAGTAAAATATGCCTTGATCAGTGCCCAGCGATGCATGATATGCCAAGGAGATATTGCTAGGTACCGGGAGCAAGCCAGTGATACAGCGAATTATGGGAAAGCACGCAGTTGGTACCTGAAGGCCCAGCACATTGCTCCCAAGAATGGGCGCCCCTATAACCAGTTGGCTTTGCTGGCAGTGTATACGAGGAGGAAGCTTGACGCTGTCTATTACTATATGCGCAGTTTAGCTGCCAGCAACCCTATCCTGACTGCCAAGGAGAGTCTCATGAGCTTGTTTGAAGAGACCAAGCGGAAGGCAGAACAGATGGAAAAGAAGCAACATGAGGAATTTGACCTGAGCCCTGACCAGTGGCGGAAAGGAAAGAAGTCTACTTTCCGGCATGTTGGAGATGACACCACTCGCCTGGAGATCTGGATTCATCCATCCCATCCACGGTCTTCCCAGGGCACTGAGTCTGGGAAGGATTCTGAGCAAGAGAATGGGCTGGGCAGCCTGAGTCCCAGTGATCTGAACAAAAGGTTCATCCTCAGTTTTCTCCATGCCCATGGGAAGCTGTTTACCCGGATTGGGATGGAGACATTCCCTGCAGTGGCTGAGAAGGTCCTCAAGGAGTTCCAGGTGTTACTGCAGCACAGCCCCTCTCCCATTGGAAGTACCCGCATGCTGCAGCTTATGACCATCAATATGTTTGCAGTACACAACTCCCAGCTGAAAGACTGCTTCTCGGAGGAGTGCCGCTCTGTGATCCAGGAACAAGCCGCAGCTCTGGGCTTGGCCATGTTTTCTCTACTGGTCCGCCGCTGCACCTGCTTACTTAAGGAGTCCGCCAAAGCTCAGCTGTCCTCTCCTGAGGACCAGGATGACCAAGACGACATCAAGGTGTCTTCCTTTGTCCCGGACCTGAAGGAGCTGCTCCCCAGTGTCAAAGTCTGGTCAGATTGGATGCTCGGCTACCCGGACACCTGGAATCCTCCTCCCACATCCCTGGATCTGCCCTCGCATGTTGCTGTGGATGTATGGTCGACGCTGGCTGATTTCTGTAACATACTGACTGCAGTGAATCAGTCTGAGGTGCCACTGTACAAGGACCCGGATGATGACCTCACCCTTCTTATCCTGGAAGAGGATCGGCTTCTCTCGGGCTTTGTCCCCTTGCTGGCTGCCCCTCAGGACCCCTGCTACGTGGAGAAAACCTCGGATAAGGTTATTGCAGCTGACTGCAAAAGGGTCACAGTGCTGAAGTATTTTCTGGAAGCCCTTTGTGGACAAGAAGAGCCTCTGCTGGCATTCAAGGGTGGAAAGTATGTGTCAGTGGCACCCGTCCCAGACACCATGGGAAAGGAAATGGGAAGCCAAGAGGGAACACGACTGGAAGATGAGGAGGAGGATGTGGTGATTGAAGACTTTGAGGAAGATTCAGAGGCTGAAGGCAGCGGAGGCGAGGATGACATCAGGGAGCTTCGGGCCAAGAAGCTGGCTCTGGCCAGGAAGATAGCTGAGCAGCAGCGTCGCCAGGAAAAGATCCAGGCTGTCCTGGAGGACCACAGTCAGATGAGGCAGATGGAGCTCGAAATCAGACCTTTGTTCCTCGTACCAGACACCAACGGCTTCATTGACCACCTGGCCAGTCTGGCGCGGCTGCTGGAGAGCAGGAAGTACATCCTGGTGGTGCCCCTCATCGTGATCAATGAGCTGGACGGCCTGGCCAAGGGGCAGGAGACAGACCACCGGGCTGGGGGCTACGCCCGTGTGGTACAAGAGAAGGCCCGCAAGTCCATCGAGTTCCTCGAGCAGCGATTCGAGAGTCGGGACTCTTGCCTGCGAGCCCTGACCAGCCGTGGCAATGAACTCGAATCCATCGCCTTCCGCAGTGAGGACATCACTGGCCAGCTGGGTAACAACGATGATCTCATCCTGTCCTGCTGCCTCCACTACTGCAAAGACAAGGCTAAGGACTTCATGCCCGCCAGCAAAGAGGAGCCAATCCGGCTACTGCGGGAGGTGGTGCTGTTGACGGATGACCGGAACCTGCGTGTGAAGGCGCTCACAAGGAATGTTCCTGTACGGGACATCCCAGCCTTCCTCACGTGGGCCCAGGTGGGCGCGGCCGCCGACTACAAGGACCACGACGGTGACTACAAGGACCACGACATCGACTACAAGGACGACGACGACAAG**TGA**TTCTAGAGGATCATAATCAGCCATACCACATTTGTAGAGGTTTTACTTGCTTTAAAAAACCTCCCACACCTCCCCCTGAACCTGAAACATAAAATGAATGCAATTGTTGTTGTTAACTTGTTTATTGCAGCTTATAATGGTTACAAATAAAGCAATAGCATCACAAATTTCACAAATAAAGCATTTTTTTCACTGCCTCGAGCTTCCTCATCGCCGGTACCAAAAAAAAAAAAAAAAAAAAAAAAAAAAAAAAAAAAAAAAAAAAAAAAAAAAAAAAAAAAAAAAAAAAAAAAAAAAAAAATTTTTGGAAAAGCTTATCGAT

|  | T7 Promoter |  | 3’UTR |
| --- | --- | --- | --- |
|  |  |  |  |
|  | SarsCov2 Leader |  | A(80) |
|  |  |  |  |
|  | SMG6 coding sequence |  | *Cla*I recognition site |
|  |  |  |  |
|  | 3xFLAG |  |  |
